# Characteristics of changes in the functional status of the brain before and after 1,000 m all-out paddling for different levels of dragon boat athletes

**DOI:** 10.3389/fpsyg.2023.1109949

**Published:** 2023-05-23

**Authors:** Qianqian Wu, Hongke Jiang, Changzhuan Shao, Yan Zhang, Wu Zhou, Yingying Cao, Jing Song, Bing Shi, Aiping Chi, Chao Wang

**Affiliations:** ^1^School of Sports, Shaanxi Normal University, Xi’ an, China; ^2^Physical Education Department, Shanghai Maritime University, Shanghai, China

**Keywords:** athletes, dragon boat sport, power spectrum, microstate, moderate intensity exercise

## Abstract

**Purposes:**

Dragon boat is a traditional sport in China, but the brain function characteristics of dragon boat athletes are still unclear. Our purpose is to explore the changing characteristics of brain function of dragon boat athletes at different levels before and after exercise by monitoring the changes of EEG power spectrum and microstate of athletes before and after rowing.

**Methods:**

Twenty-four expert dragon boat athletes and 25 novice dragon boat athletes were selected as test subjects to perform the 1,000 m all-out paddling exercise on a dragon boat dynamometer. Their resting EEG data was collected pre- and post-exercise, and the EEG data was pre-processed and then analyzed using power spectrum and microstate based on Matlab software.

**Results:**

Post-Exercise, the Heart Rate peak (HR peak), Percentage of Heart Rate max (HR max), Rating of Perceived Exertion (RPE), and Exercise duration of the novice group were significantly higher than expert group (*p* < 0.01). Pre-exercise, the power spectral density values in the *δ*, *α*1, *α*2, and *β*1 bands were significantly higher in the expert group compared to the novice group (*p* < 0.05). Post-exercise, the power spectral density values in the *δ*, *θ*, and *α*1 bands were significantly lower in the expert group compared to the novice group (*p* < 0.05), the power spectral density values of *α*2, *β*1, and *β*2 bands were significantly higher (*p* < 0.05). The results of microstate analysis showed that the duration and contribution of microstate class D were significantly higher in the pre-exercise expert group compared to the novice group (*p* < 0.05), the transition probabilities of A → D, C → D, and D → A were significantly higher (*p* < 0.05). Post-exercise, the duration, and contribution of microstate class C in the expert group decreased significantly compared to the novice group (*p* < 0.05), the occurrence of microstate classes A and D were significantly higher (*p* < 0.05), the transition probability of A → B was significantly higher (*p* < 0.05), and the transition probabilities of C → D and D → C were significantly lower (*p* < 0.05).

**Conclusion:**

The functional brain state of dragon boat athletes was characterized by expert athletes with closer synaptic connections of brain neurons and higher activation of the dorsal attention network in the resting state pre-exercise. There still had higher activation of cortical neurons after paddling exercise. Expert athletes can better adapt to acute full-speed oar training.

## 1. Introduction

The dragon boat race is a traditional Chinese sport with a history of more than 2,000 years. At present, the dragon boat race has become an international competition and many countries have professional dragon boat teams (Ho et al., 2009). The dragon boat athletes generally consist of 20 paddlers, a drummer and a flag bearer. Races are normally over distances ranging from 200 to 2,000 m. The dragon boat sport requires to maintaining the speed and ability to row during the race, and the athletes need to have good endurance, strength, and explosive power. Studies have shown that athletes’ post-exercise blood lactate concentrations increase as the distance of the race increases (Tesch et al., 1976). Ho et al. (2013) tested 11 male national dragon boat athletes from Japan in simulated 200 and 500 m races and showed that the aerobic energy supply ratio reached more than 50% in both cases. The above research shows that dragon boat sport is a water sports event which mainly involves half body exercise and aerobic energy consumption. In fact, many coaches select paddlers based on aerobic capacity (Fry and Morton, 1991; Singh et al., 1995). As a team event, dragon boat athletes should not only have excellent physical fitness, but also have to keep a high degree of attention. Long-term systematic training may induce the corresponding changes in the neurophysiology of dragon boat athletes, but there is still a lack of research in this aspect. In addition, short time acute training will have what kind of impact on the dragon boat athletes’ brain state? This is also an urgent problem.

Electric current generated by continuous firing of neurons in the brain is recorded by an Electroencephalogram (EEG) (Dressler et al., 2004). As a kind of bioelectrical signal, EEG contains abundant physiological information. The resting EEG can reflect the spontaneous activity of the brain when the brain is quiet, relaxed and does not perform specific tasks. With the development of cross-disciplines, EEG has been gradually applied to the field of sports. EEG can monitor the function state of the central nervous system (Müller-Putz, 2020), and EEG analysis can be used to assess the nervous system function of athletes and explore the plasticity of the brain induced by sports intervention.

Power spectrum and microstate are two common and well-established methods for EEG signal analysis. Power spectrum analysis mainly studies the characteristics of the signal in each frequency band, and the power spectrum values in different bands usually contain different physiological information (Pathania et al., 2021). Normal healthy people have relatively stable EEG power spectrum characteristics in the quiet state, while exercise causes changes in neural activity in the brain, and brain activation varies with different training parameters such as exercise level, exercise intensity, and exercise mode (Schneider et al., 2009; Brümmer et al., 2011a,b). Intensive training of motor skills over time leads to stronger connections between neurons in the brain, and athletes have an advantage in behavioral performance, with studies showing that expert golfers have higher *α* band activity than novices, and their ability to process attention and sensory information more economically and efficiently (Baumeister et al., 2008). Long-term sports training can promote the close connection between brain neurons. The power spectral density values of *α*, *β*, and *γ* bands of professional football players in resting state are significantly higher than those of ordinary professional sports college students, suggesting that long-term football training can improve the athletes’ internal concentration (Li et al., 2022). In addition, the percentage of *α* frequency of high-level athletes in the state of closed eyes and quiet is significantly higher than that of ordinary athletes (Yu et al., 2021). Long-term sports training can also lead to significant changes in brain structure. Studies have shown that the cerebral cortex structure of national short track speed skaters significantly increases in the cortical thickness of the left precuneus, left parietal lobe and right superior frontal gyrus (Wei et al., 2020), and professional basketball players have more active activities in the inferior parietal lobule and inferior frontal gyrus during movement preparation (Wu et al., 2013). Long-term, high-intensity physical exercise also drives up the frequency values of the *δ*, *θ*, and *β* bands (Lardon and Polich, 1996). EEG microstate, on the other hand, a multivariate method for measuring time-varying multivariate topologies with good sensitivity, represents the transient functional state of the brain (60–120 ms) with high temporal resolution and certain spatial organization information (Michel and Koenig, 2018), which is of great value for studying large-scale brain networks (Croce et al., 2022). A 4-cluster microstate model has been identified in multiple studies: A, B, C, and D, which represent the activation of different neurons (Koenig et al., 1999). The microstate parameters mainly include duration, occurrence, coverage and transition probability, which can explain the overall brain activity (Lehmann et al., 2005). Microstate studies have shown that the nervous system activity of excellent archers in resting state changes rapidly, with enhanced neural plasticity and efficiency (Gu et al., 2022). The research of Spring et al. (2017) shows that the average duration and coverage of class C increase after endurance exercise, and the transfer probability of other microstate types to class C movement increases, highlighting the significant role of network in sports.

There are great differences in the training characteristics of different sports. Dragon boat sport is a medium- to high-intensity racing sport that requires strong physical strength and muscle strength. Since athletes need to control their posture when rowing, they also have high requirements on their endurance and control ability. Good upper body endurance can help the athlete to paddle fast and continuously and finish the race at the best speed. Previous studies mostly focused on the development of dragon boat sport (Li, 2009) and biomechanical analysis of dragon boat rowing (Ho et al., 2009), etc., but few studies explored deeply the changes in the body characteristics brought by dragon boat training from the perspective of neurophysiology. EEG studies related to other sports have shown that acute exercise induces a significant increase in alpha waves in frontal location (Schneider et al., 2009) and may also increase overall concussive activity in the brain (Ciria et al., 2018), which is associated with cortical activation and enhanced functionality. Motion is closely related to prominence network (Spring et al., 2017), and may also be related to attention network corresponding to microstate D (Zhang et al., 2022). But how these findings relate to the changes in neural activity associated with dragon boat racing needs further research. Therefore, in this study, we first recorded EEG changes of dragon boat athletes in a quiet state with their eyes closed, and then recorded the EEG signals of two groups of subjects after acute moderate intensity 1,000 m training. This paper intends to use power spectrum and microstate analysis methods to explore the differences in neurophysiological characteristics between expert dragon boat athletes who have received long-term systematic training and novice athletes from different perspectives, as well as the effects of exercise intervention on the brain neural activity of the two groups of subjects. It is expected that this study can provide some reference for the training methods of dragon boat sport, and provide some theoretical basis for the benefits of long-term and acute sports to people. The hypothesis of this study is that compared with novice dragon boat athletes, the brain neuroplasticity of expert dragon-boat athletes is enhanced after long-term training and moderate intensity acute exercise can effectively activate the cerebral cortex.

## 2. Methods

### 2.1. Research subjects

Subjects in this study were selected from male students in a physical education college of a university, and the screening criteria were as follows: (1) Excellent physical condition, no cardiovascular disease, and other serious physical diseases; (2) Good mental status, no brain injury, no alcohol dependence or drug dependence, no family history of mental illness; (3) No recent physical injury or overexertion; (4) Novice group were dragon boat sports enthusiasts with no athletic rating, and the expert group was required to have participated in national high-level dragon boat competitions with the training period should be at least 2 years; and (5) All were right-handed. A total of 25 subjects were included in the novice group and 24 subjects were included in the expert group after screening. We explained the procedure of this experiment to all subjects, who voluntarily participated in this experiment and signed informed consent prior to the experiment. The experiment was approved by the Academic Ethics Committee of Shaanxi Normal University (number: 202116012), in accordance with the Declaration of Helsinki. The basic information about the subjects is shown in Table 1.

**Table 1:** Basic information about the subjects.

Test index	Results	
	Novice group (*n* = 25)	Expert group (*n* = 24)
Age (years)	19.72 ± 1.08	20.46 ± 0.93
Height (cm)	176.24 ± 5.57	183.92 ± 5.71
Weight (kg)	73.64 ± 9.53	85.42 ± 9.26
BMI (kg/m^2^)	23.65 ± 2.40	25.19 ± 1.70
Years of training (years)	0.74 ± 0.40	4.79 ± 2.54^**^

BMI, body mass index. There were no significant differences in age, height, weight and BMI between the novice group and expert group, and there were significant differences between years of training (Independent sample *T*-test). **indicates *p* < 0.01.

### 2.2. Dragon boat 1,000 m training process

**Figure 1:**
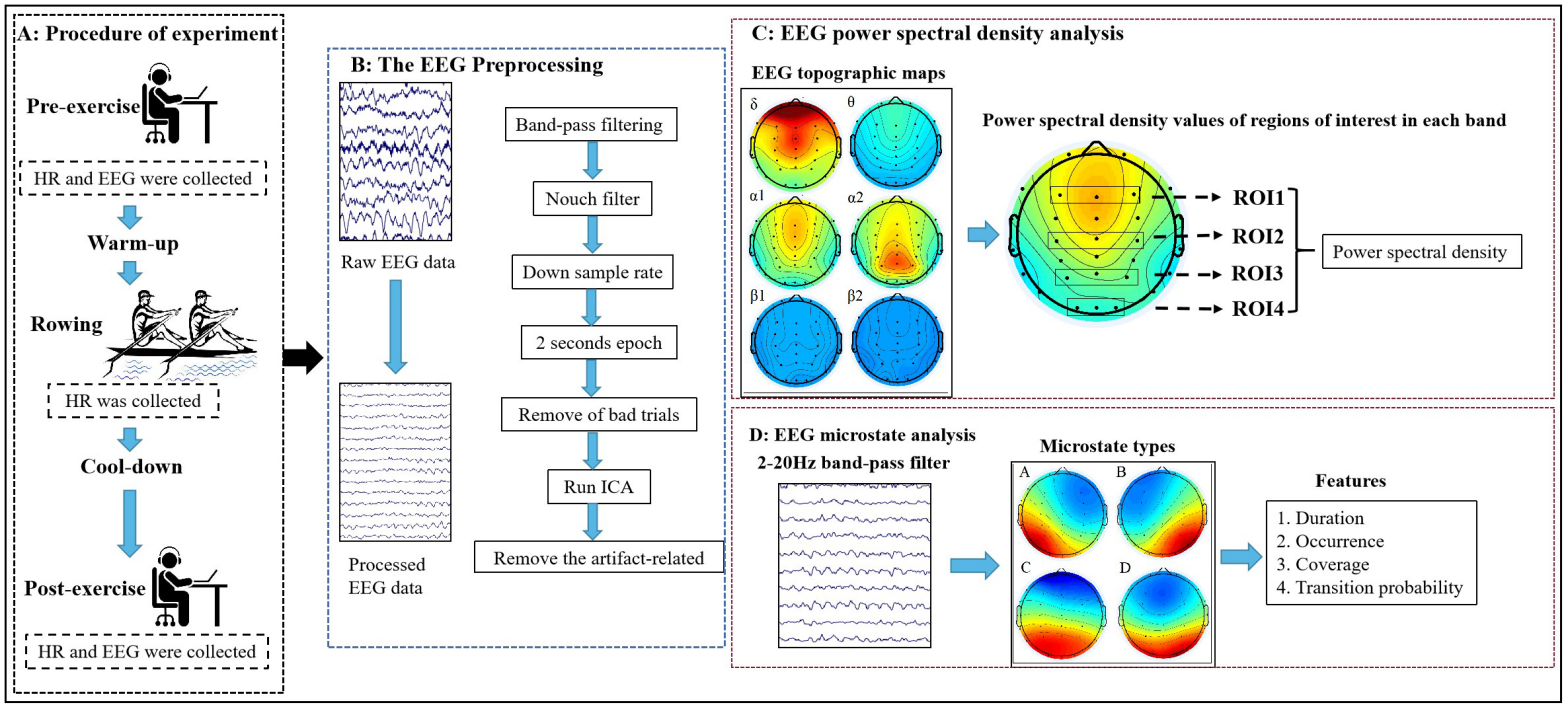
The procedure of experimental and EEG data processing flow. **(A)** Procedure of experiment; **(B)** The EEG Preprocessing; **(C)** EEG power spectral density analysis; **(D)** EEG microstate analysis. HR, Heart Rate; ROI, regions of interest.

In this experiment, a D1-M dragon boat dynamometer (KayakPro, Miami, FL, United States) was used for indoor testing. The dynamometer ranges in intensity from 0 to 9 levels, with windbox resistance increased as the number of levels increases. Subjects were required to exercise at full intensity for 1,000 m with 5 levels of windbox resistance in order to complete the target task in the shortest possible time. Throughout the test, subjects wore heart rate monitors (GT9-X, ActiLife, Pensacola, FL, United States) to collect heart rate data. Resting heart rate and EEG data before and after exercise were collected while the subjects sat still. Before the start of the experiment, subjects were informed in advance that they would not be able to perform moderate to high intensity strenuous exercise, drink alcohol, or take stimulants for 48 h. The procedure of experimental was shown in Figure 1.

### 2.3. EEG signal acquisition

EEG activity data were recorded under 32 channels according to the 10/20 international electrode placement system (Brain Vision Recorder; Neuroscan, United States). Since EEG signals are weak and easily perturbed by external signals, the experiments were performed in a quiet room with appropriate temperature and light. Subjects were first washed to reduce the scalp resistance before data acquisition, and were instructed to keep their eyes closed and awake at all times during the test, avoiding swallowing, eye movements, and leg wiggles to reduce artifacts. The EEG signal acquisition time was 6 min, the sampling frequency was 1,024 Hz, and the resistance between all electrodes and the scalp was reduced to <5 kΩ. The EEG signal acquisition and analysis were shown in Figure 1.

### 2.4. EEG data preprocessing

To analyze the EEG data in the subject’s closed-eye state, the acquired resting-state EEG data need to be pre-processed with the open-source toolbox EEGLAB (Version R2019a, MathWorks, Natick, MA, United States) software in the MATLAB (Version R2021, San Diego, CA, United States) environment. First, the CNT format data was imported into EEGLAB and converted to SET format, then the channels were located and after removing the three useless electrodes of HEO, VEO and Trigger. A 0.5–45 Hz bandpass filter, as well as a 48–50 Hz notch filter, were applied to the EEG data using a finite impulse response filter. The sampling rate was reduced to 500 Hz, the bilateral mastoids (M1, M2) were used as reference electrodes, and the continuous data were split into 2 s/segment. Visually inspect the waveforms of each electrode, If a bad electrode was present in the waveform, the bad electrode was interpolated using the default EEGLAB workflow. The Independent Component Analysis (ICA) was used to correct for additional artifactual signals such as eye movements, heart activity, and muscle contractions, to identify the principal component features, and to remove interfering components (Wu et al., 2013). Finally, the processed EEG clean data were saved after removing the larger drift segments of EEG.

### 2.5. EEG power spectrum analysis

The EEG power spectral density was analyzed by MATLAB software, and the spectrum was calculated by Pwelch function. Fast Fourier transform were conducted to estimate the EEG power spectrum density of *δ* (0.8–3.8 Hz), *θ* (4–7.8 Hz), *α*1 (8–9.8 Hz), *α*2 (9.8–12.8 Hz), and *β*1 (13–20 Hz), *β*2 (20–30 Hz) frequency. Let *X*(*n*) be an EEG spectrum, and then each segment power *I_k_* and the whole segment power *P*(*f_n_*) were calculated through Equations (1) and (3).

Sampling point *n* = 0, 1, …, *N*. *N* is the total length of the data. Divided into *X_1_* (*n*), *X_2_* (*n*), …, *X_k_* (*n*), …, *X_K_* (*n*) a total of *K* data segments covering the entire data (*K* − 1) * *D* + *L* = *N*, where *D* is the step length of each window function move, *L* is the length of each data segment, *k* = 1, 2, …, *K*. Let the window function be *W*(*n*) with sampling points *n* = 0, 1, …, *L* − 1 and data segments denoted as *X_1_*(*n*)*W*(*n*), …, *X_K_*(*n*)*W*(*n*). *h* represents EEG rhythms, (e.g., *δ*, *θ*, *α*, *β*), and *E_total_* represents the total power of all EEG rhythms.

Equation (1):


$$I_{k}\left(f_{n}\right)=\frac{1}{UL}{\left|\overset{L-1}{\underset{n=0}{\sum }}X_{k}\left(n\right)W\left(n\right)\exp\left(\frac{-i2\pi fn}{L}\right)\right|}^{2}$$

*U* is as in Equation (2):


$$U=\frac{1}{L}\overset{L-1}{\underset{n=0}{\sum }}W{\left(n\right)}^{2}$$

Equation (3):


$$P\left(f_{n}\right)=\frac{1}{K}\overset{K}{\underset{k-1}{\sum }}I_{K}\left(f_{n}\right)=\frac{1}{ULK}\overset{k}{\underset{k-1}{\sum }}{\left|\overset{L-1}{\underset{n=0}{\sum }}X_{k}\left(n\right)W\left(n\right)e^{\frac{-i2\pi fn}{L}}\right|}^{2}$$

$$R\left(h\right)=\frac{P\left(f_{n}\right)}{E_{total}}$$

The increase of slow-wave *δ* band power at resting state is related to the enhancement of the plasticity of cerebral nerve (He and Lv, 2006). *θ* wave activity in frontal lobe may be related to the degree of attention concentration (Luchsinger et al., 2016). Alpha wave is the basic rhythm of human brain wave, which is related to the excitation process, alertness, and working memory of cerebral cortex neurons (Klimesch, 2012). Beta waves are related to the oscillatory synchronization of motor cortex and somatosensory cortex (Baker, 2007) and sensory-motor interaction (Lalo et al., 2007). The gamma band is considered to be the integration of sensory and motor processes in the preparation and execution of sports (Amo Usanos et al., 2020), and is related to brain memory, motor control and other functions (Uhlhaas et al., 2008). Finally, we selected six frequency bands in four regions of interest (ROI) (ROI1: frontal region F3, FZ, and F4; ROI2: central region C3, CZ, and C4; ROI3: parietal region P3, PZ, and P4; ROI4: occipital region O1, OZ, and O2) for analysis.

### 2.6. EEG microstate analysis

In microstate analysis, a multichannel EEG signal is considered as a transient topography of a series of electrical potentials. The global field power (GFP), the standard deviation of the voltage across all scalp electrodes at a given moment in time, can describe rapid changes in brain activity, and the peak position of the GFP curve indicates the moment of maximum field strength and highest topographic signal-to-noise ratio (von Wegner et al., 2018). In order to improve the signal-to-noise ratio and make the clustering results more reliable, the EEG topographic map corresponding to the local peak points of GFP was selected for feature clustering to obtain the EEG microstate. The GFP is calculated as:


$$\mathrm{GFP}=\sqrt{\frac{\Sigma_{i=1}^{N}{\left(u_{i}-\bar{u}\right)}^{2}}{N}}$$

Where *u_i_* is the voltage across an electrode, $\bar{u}$ is the average voltage across all electrodes, and *N* is the number of electrodes.

Topographic Atomize and Agglomerate Hierarchical Clustering (T-AAHC) algorithm was used in this study, which identifies clusters with similar topological structures. The GFP peak can be obtained by the above formula calculation and the curve is plotted, the EEG topography at the time point of each local peak is extracted, and these topographies are clustered by the T-AAHC algorithm according to the determined number of microstate categories, and secondary clustering is performed after obtaining the microstate template maps of all subjects’ EEG to obtain the microstate template maps at the group level (Brunet et al., 2011). After that, the similarity between microstates and topographic GEV (Global Explanation Variance) at each time point of EEG data is calculated, and the time points corresponding to each type of microstate are labeled, so that the original EEG signal becomes a time series with four classes of microstates alternately transformed so that the temporal parameters of interest can be extracted and analyzed for differences (Seitzman et al., 2017). The temporal parameters calculated in this study are: (1) Duration: the average duration of a microstate to maintain stability in a certain time; (2) Occurrence: the number of times a particular microstate category occurs per second; (3) Contribution: the percentage of a microstate’s occurrence time to the total time; and (4) Transition probability: the percentage of the number of transitions between different microstates to the total number of transitions.

### 2.7. Statistics analysis

SPSS 23.0 (IBM SPSS Statistics, United States) software was used to analyze the data obtained in this study. The Shapiro–Wilk test was used to determine whether the data conform to the normal distribution. If the data did not conform to the normal distribution, non-parametric test was used, and parametric test was used for the data conforming to the normal distribution. Independent sample *T*-test was conducted for the four indicators (HR peak, Percentage of HR max (%), RPE, and Exercise duration) of the two groups of athletes post-exercise. For power spectral density analysis, a three-factor mixed design repeated-measures analysis of variance (ANOVA) of 2 (group) × 2 (time) × 4 (ROI) pre- and post-exercise were performed on the 6 frequency bands of the two groups. In the microstate analysis, the duration, occurrence, and contribution of microstate parameters pre- and post-exercise were analyzed by repeated-measures ANOVA with a three-factor mixed design of 2 (group) × 2 (time) × 4 (map). The Mann–Whitney *U*-test was performed for the transition probability pre- and post-exercise between expert and novice groups, respectively, and the Wilcoxon signed-rank test was performed pre- and post-exercise within the group. Mauchly’s spherical hypothesis test was used in repeated-measures ANOVA to determine if the spherical hypothesis was satisfied. If necessary, the Greenhouse–Geisser method was used to correct for those that did not satisfy the spherical hypothesis test, and the Bonferroni method was used to correct for post-hoc pairwise comparisons. The results of the data were expressed as “mean ± standard deviation,” and *p* < 0.05 was considered as the criterion for significant difference, and partial *η^2^* was used to calculate the effect size.

## 3. Results

### 3.1. Comparison of athletes’ post-exercise performance

The performance of the two groups of dragon-boat athletes with different levels post-exercise is shown in the Table 2.

**Table 2:** Movement performance after completing 1,000 m dragon boat dynamometer (Mean ± SD).

Physiological indexes	Novice group	Expert group
HR peak (b/min)	178.34 ± 6.01	170.37 ± 5.21^**^
Percentage of HR max (%)	89.06 ± 3.20	85.38 ± 2.63^**^
RPE	18.04 ± 0.87	17.13 ± 0.67^**^
Exercise duration (s)	306.56 ± 31.69	259.75 ± 28.02^**^

HR, heart rate; HR peak, heart rate peak; HR max, heart rate max; RPE, rating of perceived exertion; Percentage of HR max (%) = HR peak/HR max, >90%: super-intensity exercise, 80%–90%: high-intensity exercise, 60%–80%: moderate-intensity exercise, <60% small-intensity exercise; *indicates *p* < 0.05; **indicates *p* < 0.01.

We compared the HR peak, Percentage of HR max (%), RPE, and Exercise duration of the two groups of athletes. The results showed that there were significant differences between the two groups of athletes post-exercise, and the four indexes of the novice group were higher than those of the expert group (*p* < 0.01).

### 3.2. Power spectrum analysis results

**Figure 2:**
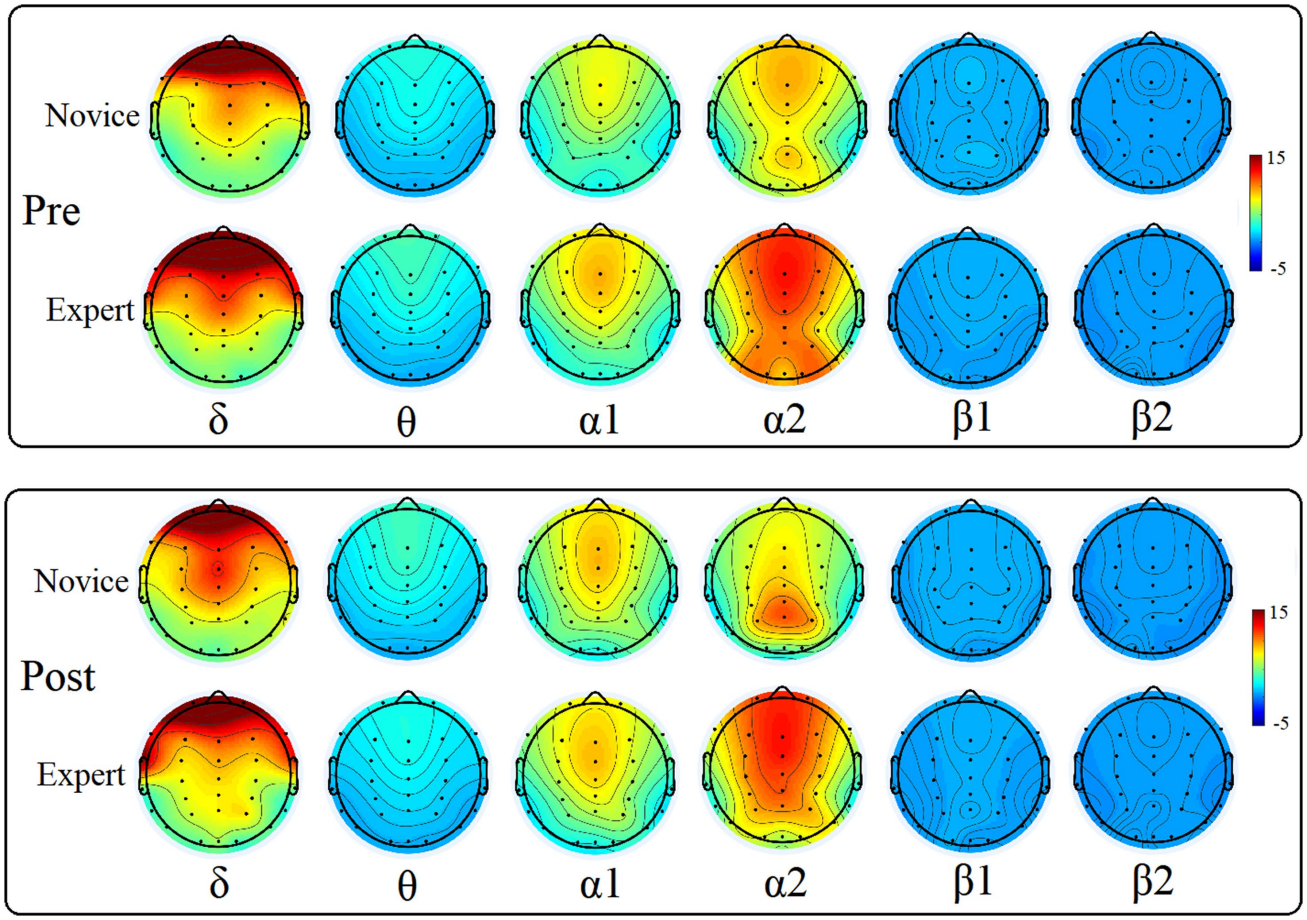
Power spectrum topographic maps of different bands pre- and post-exercise of the two groups of subjects.

The EEG data of two groups of dragon boat athletes with different levels pre- and post-exercise were analyzed by power spectrum analysis, and the EEG topographic map of each band was obtained, as shown in Figure 2, and the power spectrum density value is shown in Table 3.

**Table 3:** Logarithmic conversion values of power spectral density pre- and post-exercise (Mean ± SD).

Band	ROI	Pre		Post		Values of *p*			
		Novice	Expert	Novice	Expert	Novice vs. expert		Pre vs. post	
						Pre	Post	Novice	Expert
*δ*	ROI1	2.27 ± 0.23	2.53 ± 0.34	2.36 ± 0.30	2.29 ± 0.42	0.003^**^	0.484	0.266	0.004^**^
	ROI2	1.93 ± 0.35	2.17 ± 0.33	2.19 ± 0.43	1.88 ± 0.41	0.015^*^	0.012^*^	0.003^**^	0.001^**^
	ROI3	1.70 ± 0.45	1.76 ± 0.47	1.80 ± 0.53	1.65 ± 0.62	0.627	0.369	0.391	0.291
	ROI4	1.44 ± 0.51	1.32 ± 0.68	1.54 ± 0.35	1.07 ± 0.54	0.469	0.001^**^	0.356	0.015^*^
*θ*	ROI1	0.86 ± 0.38	1.05 ± 0.46	0.99 ± 0.43	0.83 ± 0.39	0.117	0.163	0.152	0.023^*^
	ROI2	0.60 ± 0.44	0.74 ± 0.47	0.88 ± 0.46	0.56 ± 0.39	0.311	0.013^*^	0.013^*^	0.118
	ROI3	0.16 ± 0.42	0.26 ± 0.55	0.33 ± 0.62	0.28 ± 0.46	0.508	0.721	0.197	0.881
	ROI4	0.19 ± 0.49	0.25 ± 0.54	0.18 ± 0.51	0.25 ± 0.52	0.664	0.646	0.982	0.993
*α*1	ROI1	1.31 ± 0.66	1.43 ± 0.48	1.78 ± 0.83	1.37 ± 0.53	0.462	0.044^*^	0.003^**^	0.691
	ROI2	1.31 ± 0.81	1.36 ± 0.55	1.67 ± 0.82	1.74 ± 0.41	0.800	0.702	0.051	0.043^*^
	ROI3	0.99 ± 0.97	1.54 ± 0.71	1.46 ± 0.91	1.55 ± 0.68	0.009^**^	0.677	0.025^*^	0.954
	ROI4	0.85 ± 0.76	0.63 ± 1.09	0.63 ± 1.04	0.97 ± 0.89	0.412	0.214	0.455	0.260
*α*2	ROI1	1.91 ± 0.72	2.23 ± 0.66	1.95 ± 0.40	2.21 ± 0.51	0.108	0.045^*^	0.789	0.907
	ROI2	1.71 ± 0.72	2.14 ± 0.63	1.72 ± 0.87	2.16 ± 0.52	0.032^*^	0.041^*^	0.919	0.905
	ROI3	1.54 ± 0.84	2.17 ± 0.67	1.78 ± 1.18	2.16 ± 0.57	0.006^**^	0.160	0.190	0.963
	ROI4	1.31 ± 1.20	2.00 ± 0.85	1.55 ± 0.50	1.60 ± 0.63	0.024^*^	0.751	0.278	0.088
*β*1	ROI1	0.27 ± 0.42	0.18 ± 0.45	0.39 ± 0.37	0.23 ± 0.41	0.448	0.167	0.207	0.554
	ROI2	0.40 ± 0.39	0.26 ± 0.50	0.49 ± 0.38	0.23 ± 0.41	0.284	0.049^*^	0.368	0.922
	ROI3	0.61 ± 0.54	0.24 ± 0.59	0.25 ± 0.42	0.42 ± 0.61	0.025^*^	0.262	0.008^**^	0.194
	ROI4	0.82 ± 0.58	0.37 ± 0.57	0.92 ± 0.47	0.67 ± 0.57	0.008^**^	0.093	0.235	0.002^**^
*β*2	ROI1	0.89 ± 0.22	0.86 ± 0.26	0.89 ± 0.33	0.70 ± 0.43	0.570	0.090	0.924	0.084
	ROI2	1.01 ± 0.27	0.95 ± 0.35	1.01 ± 0.28	0.87 ± 0.42	0.436	0.177	0.926	0.448
	ROI3	1.21 ± 0.38	1.13 ± 0.49	0.89 ± 0.39	0.88 ± 0.52	0.589	0.896	0.011^*^	0.035^*^
	ROI4	1.35 ± 0.53	1.19 ± 0.55	1.31 ± 0.37	0.71 ± 0.58	0.290	0.000^**^	0.720	0.001^**^

ROI, regions of interest; ROI1, frontal region; ROI2, central region; ROI3, parietal region; ROI4, occipital region; *indicates *p* < 0.05; **indicates *p* < 0.01.

A 2 (group) × 2 (time) × 4 (ROI) mixed design ANOVA was performed for the *δ* band. The main effect of ROI was significant [*F*(2.151, 101.095) = 222.528, *p* < 0.001, *η*^2^ = 0.826]. The interaction between time and group was significant [*F*(1, 47) = 10.832, *p* = 0.002, *η*^2^ = 0.187]. The results of the simple effect analysis showed that the expert group had significantly lower values of the power spectral density post-exercise than the novice group, and that the expert group had significantly lower values of the power spectral density post-exercise than pre-exercise. The interaction between ROI and group was significant [*F*(2.151, 101.095) = 7.555, *p* = 0.001, *η*^2^ = 0.138]. The power spectral density values in the ROI4 were significantly lower in the expert group than in the novice group. The interaction margin of the group, time, and ROI was significant [*F*(2.165, 101.734) = 2.661, *p* = 0.050, *η*^2^ = 0.054]. The results of the simple effect analysis showed that the power spectral density values in the ROI1 and ROI2 regions were significantly higher in the pre-exercise expert group than in the novice group. Post-exercise, the power spectral density values in the ROI2 and ROI4 of the expert group were significantly lower than those of the novice group. In the novice group, the power spectral density values in the ROI2 were significantly higher post-exercise than pre-exercise. In the expert group, the power spectral density values of ROI1, ROI2, and ROI4 were significantly lower post-exercise than pre-exercise. Other main effects and interaction effects were not significant.

A 2 (group) × 2 (time) × 4 (ROI) mixed design ANOVA was performed for the *θ* band. The main effect of ROI was significant [*F*(1.669, 78.432) = 293.226, *p* < 0.001, *η*^2^ = 0.862]. The edge of interaction between time and ROI was significant [*F*(2.030, 95.413) = 2.665, *p* = 0.050, *η*^2^ = 0.054]. The interaction between group, time, and ROI was significant [*F*(2.030, 95.413) = 7.549, *p* = 0.001, *η*^2^ = 0.138]. The results of the simple effect analysis showed that the power spectral density values of the expert group in the ROI2 were significantly lower post-exercise compared to the novice group. In the novice group, the power spectral density values in the ROI2 was significantly higher post-exercise than pre-exercise. In the expert group, the power spectral density values in the ROI1 were significantly lower post-exercise than pre-exercise. Other main effects and interaction effects were not significant.

A 2 (group) × 2 (time) × 4 (ROI) mixed design ANOVA was performed for the *α*1 band. The main effect of ROI was significant [*F*(1.524, 71.626) = 60.260, *p* < 0.001, *η*^2^ = 0.562]. The interaction between ROI and group was significant [*F*(1.524, 71.626) = 4.601, *p* = 0.021, *η*^2^ = 0.089]. The interaction between group, time, and ROI was significant [*F*(1.279, 60.109) = 5.613, *p* = 0.015, *η*^2^ = 0.107]. The results of simple effect analysis showed that the power spectral density values in the ROI3 of the expert group were significantly higher than that of the novice group pre-exercise. And the power spectral density values in the ROI1 of the expert group were significantly lower than that of the novice group post-exercise. In the novice group, the power spectral density values in the ROI1 and ROI3 were significantly higher post-exercise than pre-exercise. In the expert group, the power spectral density values in the ROI4 were significantly higher post-exercise than pre-exercise. Other main effects and interaction effects were not significant.

A 2 (group) × 2 (time) × 4 (ROI) mixed design ANOVA was performed for the *α*2 band. The main effect of the group was significant [*F*(1, 47) = 6.277, *p* = 0.016, *η*^2^ = 0.118], and the power spectral density values of the expert group were significantly higher than that of the novice group. The main effect of ROI was significant [*F*(2.293, 107.751) = 22.171, *p* = 0.000, *η*^2^ = 0.321]. The interaction between group, time, and ROI was significant [*F*(1.616, 75.964) = 3.634, *p* = 0.015, *η*^2^ = 0.072]. The results of the simple effect analysis showed that the power spectral density values in the ROI2, ROI3, and ROI4 were significantly higher in the pre-exercise expert group than in the novice group. Post-exercise, the power spectral density values in the ROI3 and ROI4 were significantly higher in the expert group than in the novice group. Other main effects and interaction effects were not significant.

A 2 (group) × 2 (time) × 4 (ROI) mixed design ANOVA was performed for the *β*1 band. The main effect of ROI was significant [*F*(1, 47) = 6.277, *p* = 0.016, *η*^2^ = 0.118]. The interaction between ROI and group was significant [*F*(1.712, 80.476) = 4.201, *p* = 0.023, *η*^2^ = 0.082]. The power spectral density values in the ROI4 were significantly higher in the expert group than in the novice group. The interaction between time and ROI was significant [*F*(2.186, 102.744) = 9.548, *p* < 0.001, *η*^2^ = 0.169], and the power spectral density values in the ROI4 were significantly lower post-exercise than pre-exercise. The interaction between group, time, and ROI was significant [*F*(2.186, 102.744) = 13.939, *p* < 0.001, *η*^2^ = 0.229]. The results of simple effect analysis showed that the power spectral density values in the ROI3 and ROI4 were significantly higher in the expert group than in the novice group pre-exercise. Post-exercise, the power spectral density values in the ROI2 were significantly higher in the expert group than in the novice group. In the novice group, the power spectral density values of the ROI3 post-exercise were significantly higher than that of pre-exercise. In the expert group, the power spectral density values of the ROI4 were significantly lower post-exercise than pre-exercise. Other main effects and interaction effects were not significant.

A 2 (group) × 2 (time) × 4 (ROI) mixed design ANOVA was performed for the *β*2 band. The main effect of the group was significant [*F*(1, 47) = 4.597, *p* = 0.037, *η*^2^ = 0.089], and the power spectral density values of the expert group were significantly higher than that of the novice group. The main effect of time was significant [*F*(1, 47) = 6.584, *p* = 0.014, *η*^2^ = 0.123], and the power spectral density values were significantly higher post-exercise than pre-exercise. The main effect of ROI was significant [*F*(1.944, 91.357) = 24.064, *p* < 0.001, *η*^2^ = 0.339]. The interaction between ROI and group was significant [*F*(1.944, 91.357) = 8.522, *p* < 0.001, *η*^2^ = 0.153]. The power spectral density values in the ROI4 were significantly higher in the expert group than in the novice group. The interaction between time and ROI was significant [*F*(1.858, 87.306) = 5.871, *p* = 0.005, *η*^2^ = 0.111]. The power spectral density values in the ROI3 and ROI4 were significantly higher in the post-exercise than in the pre-exercise. The interaction between group, time, and ROI was significant [*F*(1.858, 87.306) = 4.120, *p* = 0.022, *η*^2^ = 0.081]. The results of the simple effect analysis showed that the post-exercise ROI4 power spectral density values were significantly higher in the expert group than in the novice group. In the novice group, the power spectral density values in the ROI3 were significantly higher post-exercise than pre-exercise. In the expert group, the power spectral density values in the ROI3 and ROI4 were significantly higher post-exercise than pre-exercise. Other main effects and interaction effects were not significant.

### 3.3. Comparison of microstate brain topography

**Figure 3:**
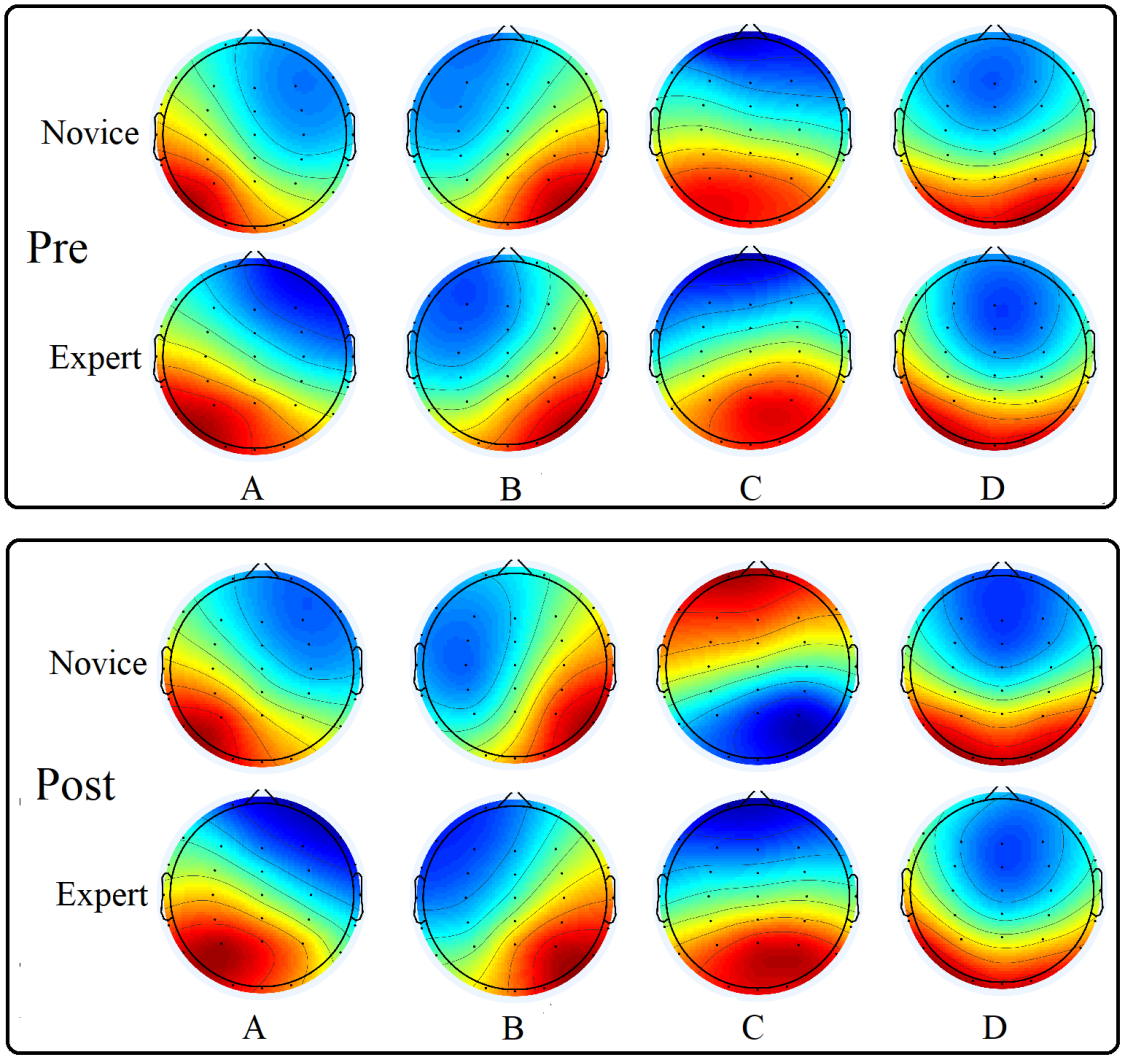
Four types of microstate topographic maps of the two groups of subjects pre- and post-exercise.

According to the above microstate analysis method, the EEG of the two groups of athletes pre- and post-exercise was calculated. After clustering, four classes of microstate topographic maps were obtained (Figure 3), which were recorded as class A, B, C, and D microstates. The polarity of the four microstate EEG maps obtained in this study was right anterior to left posterior, left anterior to right posterior, midline anterior to posterior, and apical to posterior. And there was no significant change pre- and post-exercise.

### 3.4. Microstate parameter analysis

A 2 (group) × 2 (time) × 4 (map) mixed design ANOVA was performed for the duration of microstate. The main effect of the map was significant [*F*(3, 141) = 5.482, *p* = 0.001, *η*^2^ = 0.104]. The interaction between the map and group was significant [*F*(3, 141) = 2.798, *p* = 0.043, *η*^2^ = 0.056]. The duration of microstate class C in the expert group was significantly lower than that in the novice group. The interaction of the group, time, and map was significant [*F*(3, 141) = 4.173, *p* = 0.007, *η*^2^ = 0.082]. The results of the simple effect analysis showed that the duration of microstate class D was significantly higher in the expert group than in the novice group pre-exercise. Post-exercise, the duration of microstate class C was significantly lower in the expert group than in the novice group. In the novice group, the duration of microstate classes C and D post-exercise was significantly higher than that of pre-exercise. Other main effects and interaction effects were not significant (Table 4).

**Table 4:** Characteristics of microstate parameters pre- and post-exercise in the two groups (Mean ± SD).

Index	Map	Pre		Post		Values of *p*			
		Novice	Expert	Novice	Expert	Novice vs. expert		Pre vs. post	
						Pre	Post	Novice	Expert
Duration	A	71.55 ± 7.81	69.78 ± 10.28	70.84 ± 8.05	69.78 ± 9.11	0.500	0.667	0.773	0.999
	B	70.03 ± 8.72	66.53 ± 8.18	68.14 ± 6.92	68.16 ± 7.75	0.155	0.991	0.385	0.462
	C	65.40 ± 6.77	64.79 ± 5.12	71.14 ± 8.61	64.54 ± 5.34	0.727	0.002^**^	0.003^**^	0.896
	D	65.40 ± 10.80	71.65 ± 8.48	70.33 ± 8.68	68.27 ± 9.04	0.029^*^	0.420	0.033^*^	0.145
Occurrence	A	3.66 ± 0.52	3.82 ± 0.38	3.57 ± 0.46	3.91 ± 0.48	0.227	0.014^**^	0.380	0.455
	B	3.68 ± 0.38	3.62 ± 0.39	3.59 ± 0.43	3.81 ± 0.50	0.631	0.112	0.484	0.126
	C	3.66 ± 0.39	3.63 ± 0.57	3.73 ± 0.40	3.55 ± 0.61	0.840	0.228	0.644	0.599
	D	3.85 ± 0.38	3.80 ± 0.50	3.58 ± 0.49	3.89 ± 0.54	0.711	0.041^*^	0.030^*^	0.477
Contribution	A	25.84 ± 3.20	26.47 ± 4.03	24.86 ± 3.66	26.87 ± 3.42	0.553	0.054	0.314	0.687
	B	25.83 ± 4.03	23.80 ± 3.45	24.08 ± 2.98	25.76 ± 3.85	0.065	0.094	0.093	0.067
	C	23.67 ± 3.76	23.37 ± 4.23	26.69 ± 3.68	22.33 ± 3.29	0.792	0.000^**^	0.010^*^	0.372
	D	23.62 ± 4.99	27.17 ± 6.27	25.49 ± 4.96	25.76 ± 5.38	0.033^*^	0.987	0.186	0.247

*indicates *p* < 0.05; **indicates *p* < 0.01.

A 2 (group) × 2 (time) × 4 (map) mixed design ANOVA was performed for the occurrence of microstate. The interaction between the map and group was significant [*F*(3, 141) = 2.873, *p* = 0.039, *η*^2^ = 0.058], and the occurrence of microstate class A in the expert group was significantly higher than that in the novice group. Other main effects and interaction effects were not significant (Table 4).

A 2 (group) × 2 (time) × 4 (map) mixed design ANOVA was performed for the contribution of microstate. The main effect of the group was significant [*F*(3, 141) = 2.972, *p* = 0.034, *η*^2^ = 0.059]. The interaction between time and group was significant [*F*(1, 47) =5.660, *p* = 0.021, *η*^2^ = 0.107], and the contribution of the pre-exercise expert group was significantly higher than that of the novice group. The interaction between the map and group was significant [*F*(3, 141) = 3.525, *p* = 0.017, *η*^2^ = 0.070]. The contribution of microstate class C was significantly lower in the expert group than in the novice group. The interaction of the group, time, and map was significant [*F*(3, 141) = 4.177, *p* = 0.007, *η*^2^ = 0.082]. The results of the simple effect analysis showed that the contribution of microstate class D was significantly higher in the pre-exercise expert group than in the novice group. Post-exercise, the contribution of microstate class C was significantly lower in the expert group than in the novice group. In the novice group, the contribution of microstate class C was significantly higher post-exercise than pre-exercise. Other main effects and interaction effects were not significant (Table 4).

Since the transition probability of microstate did not follow a normal distribution, The Mann–Whitney *U*-test was performed for the transition probability pre- and post-exercise between expert and novice groups, respectively, and the Wilcoxon signed-rank test was performed pre- and post-exercise within the group. The transition probabilities of A → D, C → D, and D → A in the expert group pre-exercise were significantly higher than those in the novice group. The transition probability of A → B in the expert group post-exercise was significantly higher than those in the novice group, and the transition probabilities of C → D and D → C were significantly lower than those in the novice group. In the novice group, the post-exercise transition probabilities of A → B and B → D were significantly lower, and the post-exercise transition probabilities of B → C, C → D, and D → C were significantly higher than those pre-exercise. In the expert group, the post-exercise transition probability of B → C was significantly higher, and the post-exercise transition probability of C → D was significantly lower than those pre-exercise (Table 5).

**Table 5:** Characteristics of microstate transition probability parameters pre- and post-exercise in two groups of subjects (Mean ± SD).

Transitions	Pre		Post		Values of *p*			
	Novice	Expert	Novice	Expert	Novice vs. expert		Pre vs. post	
					Pre	Post	Novice	Expert
A → B	8.30 ± 2.02	7.63 ± 2.04	7.41 ± 1.40	8.87 ± 2.16	0.285	0.008^**^	0.030^*^	0.106
A → C	7.58 ± 1.57	8.01 ± 1.91	7.90 ± 1.86	7.65 ± 1.84	0.704	0.936	0.382	0.424
A → D	7.99 ± 2.37	9.19 ± 2.04	8.24 ± 1.73	8.48 ± 1.89	0.023^*^	0.496	0.638	0.391
B → A	8.33 ± 1.99	7.72 ± 1.86	7.87 ± 1.44	8.47 ± 1.74	0.395	0.180	0.288	0.253
B → C	7.48 ± 1.50	7.21 ± 1.43	8.74 ± 1.73	8.68 ± 2.62	0.645	0.873	0.030^*^	0.018^*^
B → D	8.23 ± 1.55	8.17 ± 2.60	7.36 ± 1.68	8.65 ± 2.73	0.674	0.057	0.049^*^	0.241
C → A	7.49 ± 1.54	7.76 ± 1.50	7.57 ± 1.71	7.73 ± 1.88	0.936	0.575	0.830	0.920
C → B	7.86 ± 2.45	7.50 ± 1.50	8.39 ± 1.57	7.28 ± 2.00	0.509	0.097	0.183	0.819
C → D	7.52 ± 1.44	8.92 ± 2.45	8.68 ± 2.20	7.16 ± 1.71	0.030^*^	0.016^*^	0.013^*^	0.003^**^
D → A	8.04 ± 2.31	9.29 ± 1.78	8.20 ± 1.60	8.81 ± 2.09	0.018^*^	0.603	0.716	0.458
D → B	8.36 ± 1.65	8.07 ± 2.56	7.66 ± 1.51	8.35 ± 2.21	0.222	0.258	0.162	0.353
D → C	7.48 ± 1.51	8.18 ± 2.44	8.88 ± 2.11	7.27 ± 1.94	0.308	0.019^*^	0.012^*^	0.376

*indicates *p* < 0.05; **indicates *p* < 0.01.

## 4. Discussion

In this study, the dragon boat dynamometer was used to conduct a one-time moderate intensity exercise test on two groups of dragon boat athletes with different levels, and the heart rate monitor was used to monitor the athletes’ heart rate changes during the whole test. Although we set the exercise intensity as medium, athletes were required to go all out in the 1,000 m test. The HR peak and Percentage of HR max of the last two groups of athletes reached the effect of high intensity exercise, and RPE also indicated that athletes had certain exercise fatigue. At the same time, we collected the resting EEG data pre- and post-exercise, and used EEG power spectrum and microstate methods to compare and analyze the brain activity of dragon boat athletes of different levels pre- and post-exercise. The results are consistent with our hypothesis. Long-term systematic exercise training can promote closer connection between brain regions. And moderate intensity acute exercise activates the cerebral cortex. The training mode adopted in this study is more suitable for expert dragon boat athletes to carry out special endurance training of upper limbs.

### 4.1. Power spectral density analysis

The results showed that the power spectral density values of *δ*, *α*1, *α*2, and *β*1 bands in the resting state pre-exercise were significantly higher in expert group athletes than in novice group athletes. Earlier studies reported higher power spectral values in the *α*, *β*, and *γ* bands for expert soccer players than for novice athletes in the resting state, and the reason for this was related to the higher intrinsic concentration of expert soccer players (Li et al., 2022). In addition, professional curlers (Yu et al., 2021) and top-level karate players showed higher power values in the *α*1 band during quiet times (Babiloni et al., 2010). It is commonly believed that prolonged learning of expert motor skills leads to closer connections between central nerves, an increased plasticity between neurons, and expert athletes tend to excel in neural efficiency, cognitive flexibility, and cortical activation accuracy compared to non-expert athletes (Dayan and Cohen, 2011). In contrast to table tennis and badminton, which require a high attention span, dragon boating focuses more on the control of paddling posture, the quality of endurance and explosive power of the body, and the cooperation between the teams. In this study, the power spectral density values of *α* band of expert group dragon boat athletes were higher and mainly concentrated in the central, parietal, and occipital regions, suggesting that our long-term systematic dragon boat training improved the athletes’ intrinsic concentration and excellent control of body posture. The appearance of *β* band may be related to alertness or anxiety (Dzedzickis et al., 2020), and the significant increase of *β* band power spectral density in the expert group may be due to the highly alert mental state of athletes. In addition, the energy values of *δ* band of expert group dragon boat athletes in the quiet state also showed elevated, additionally indicating that long-term systematic training could enhance the connections between brain regions of expert athletes and improve the plasticity of the brain. Dragon boat training can also be further promoted to the general population. In the learning process of dragon boat sports, it can not only inherit the excellent traditional Chinese culture, improve the ability of team assistance, but also improve the neural plasticity of the brain and enhance the degree of self-concentration.

After performing moderate intensity 1,000 m training of dragon boat exercise, the results showed that the power spectral density values of *α*1 and *β*2 bands were significantly higher in both expert and novice groups compared to pre-exercise. The trend of increased *α* band power after exercise indicates an increased synchronization of cortical neuronal activity dominated by *α* band, reflecting the increased intensity of cortical neuronal metabolism (He and Lv, 2006), and the increased *β* band is considered to be an indication of increased excitability of nerve cells (Amo Usanos et al., 2020). Komiyama et al. (2021) showed that moderate-intensity acute aerobic cycling induced an increase in cortical alpha-wave activity. Fumoto et al. (2010) also showed a significant increase in the power values of high-frequency alpha-wave after exercise, but a significant decrease in the *θ* band and no significant change in the *β* band. The study by Crabbe et al. suggested that our brain activity was enhanced after exercise not only in the *α* band but also in the *δ*, *θ*, and *β* frequency bands were also increased (Crabbe and Dishman, 2004). Another study (Ciria et al., 2018) showed that the increase in brain activity after exercise was overall in the low-frequency band and was mainly concentrated in the parieto-occipital region in the high-frequency band. Previous studies have mostly confirmed that acute physical exercise increases the activity of brain neurons, activates brain mechanisms, and promotes the ability to process information (Gutmann et al., 2015). Exercise modulates brain-derived neurotrophic factor (BDNF) and induces an increase in the number of mitochondria in neurons and glial cells, thus improving brain microcirculation (Pedersen et al., 2009); acute aerobic exercise also promotes the development of neuroplasticity and selectively affects the connectivity of brain structures such as the hippocampus and motor cortex (Smith et al., 2014); and activates the sympathetic nervous system and the hypothalamic–pituitary–adrenal axis, which leads to the release of central and peripheral neurocatecholamines (Du Rietz et al., 2019). The influence of body temperature on exercise should not be ignored. Studies show that after intense exercise, the body temperature will rise, and the metabolic rate of the brain will also accelerate, the frequency of brain wave is proportional to the speed of brain metabolic process, so the increase of body temperature will lead to the increase of brain wave frequency and amplitude, brain metabolism is accelerated, which is also the main reason for the increase of alpha band cerebral electrical power after exercise (Nybo and Secher, 2004; Luchsinger et al., 2016). The increased energy values in the *α* and *β* bands in this study also further suggested that acute exercise activates brain area activity and improved cognitive function in people. As an exercise with mixed energy supply based on aerobic energy supply, special aerobic endurance training is essential. From the perspective of brain neurophysiology, the acute 1,000 m distance endurance training adopted in this study can better improve the nerve cell excitability of athletes, which can be verified from more physiological and biochemical indicators in the future. Furthermore, the influence of long-term special endurance training on the body of athletes is deeply explored.

We also noted a significant increase in *δ* and *θ* bands’ power spectral density values in the post-exercise novice group compared to the pre-exercise period. He (2003) compared the EEG topography after three exercise intensities and showed that the EEG power of slow wave increased obviously with the increase of exercise intensity. The results show that with the increase of exercise intensity, the EEG power of slow wave increases obviously with the appearance of *δ* band, which has a more profound effect on the central nervous system at moderate to high intensities. The field of sports medicine considered the appearance of *δ* and *θ* bands as a manifestation of exercise fatigue in the bioelectrical activity of the brain (Zhang et al., 1991). We hypothesize that novice athletes may consume higher amounts of energy due to constant stress-induced arousal of the central nervous system compared to expert athletes with long training sessions. The brain’s preferred energy supply comes from glucose oxidation, and direct measurements of the effect of exercise intensity on brain glucose uptake decreased the glucose metabolic rate (rGMR) in all measured cortical regions as exercise intensity increased (Kemppainen et al., 2005), contributing to the development of exercise fatigue. In addition, novice athletes have relatively weak postural control of rowing movements and expend more additional energy, so they are more susceptible to fatigue after acute training. For novice athletes, specific endurance training may need to be adjusted, such as rowing at a constant speed or variable speed training is also well worth trying.

The comparison of post-exercise EEG change characteristics between the two groups of subjects was noteworthy. The results of this study showed that the power spectral density values in the *δ*, *θ*, and *α*1 frequency bands were significantly lower in the expert group and significantly higher in the *α*2, *β*1, and *β*2 frequency bands after exercise compared to the novice group. The increase in slow wave frequency after exercise is commonly associated with fatigue generation (Zhang et al., 1991), expert dragon boat athletes have to undergo weekly high-intensity expert training and have better strength and endurance qualities, while long-term training allows them to master the correct paddling posture and have more precise control of the body. In this way, the expert group is more time and effort efficient than the novice group when the training tests is conducted under the same conditions. The slow wave frequency was significantly lower in the expert group compared to the novice group in this study, suggesting that the expert group was more adaptive under training conditions of equal intensity and distance, while the novice group showed a decreasing trend in the working capacity of the neural cells. *α* band is also involved in participating in individual arousal and attentional states, and as the amount of cortical working memory increases, the *α* rhythm increases, which can reduce the target information in working memory from extraneous external stimuli interference (Wang et al., 2017). Studies have shown that great soccer players show increased *α* ERD in the bilateral frontal lobes compared to non-athletes in cognitive task tests (Babiloni et al., 2008). We hypothesize that the power spectral density values in the *α*2 band are significantly increased in the expert group of dragon boat athletes compared to the novice group, most likely because the cortical motor areas of expert athletes are more sensitive and accurate to motor stimuli. The increase of beta-wave means the increase of excitability of cerebral cortex (He, 2003). However, some studies have shown that the *β* ERD of professional shooting athletes is lower than that of the novice group (Haufler et al., 2000), which may be related to the sports items and exercise intensity of the study.

### 4.2. Microstate analysis

Microstate analysis methods can be used to obtain microstate time series by computing multi-channel EEG, which can greatly reflect the characteristics of electrical activity in both the time and spatial domains of the whole brain (Lehmann et al., 1987). EEG microstate is also known as “atoms of the mind,” and microstate time series are abundant in potentially neurophysiologically relevant parameters (Britz et al., 2010). Each microstate class corresponds to a resting-state large-scale brain network obtained from blood-oxygen-level dependent (BLOD), and microstates A, B, C, and D correspond to language processing, visual networks, cognitive control networks, and attention-related resting-state networks, respectively (Custo et al., 2017). Many studies have used resting-state EEG microstates as biomarkers for diagnosing diseases (de Bock et al., 2020), and some scholars have applied them to the evaluation of brain load and brain fatigue (Guan et al., 2022), but the application in the field of exercise is still in its infancy.

Microstate analysis can partially compensate for the deficiency of EEG in identifying spatial resolution and analyzing large-scale brain network abnormalities. In this study, we performed clustering operation to obtain four classes of microstates based on the EEG state at the peak moment of the GFP curve, and then calculated the duration, occurrence, contribution, and transition probability. The results of the study showed significantly higher duration and contribution of microstate class D in the expert group in the quiet state compared to the novice group, which is more consistent with the study by Gu et al. (2022). They recorded the EEG of 13 national and provincial archery athletes in the resting state as well as during aiming, respectively, and the results showed that athletes with higher levels of microstate class D in the resting state had higher duration, occurrence, and contribution, and showed higher neural efficiency. From the neurofunctional plasticity perspective, researchers have suggested that continuous learning and memory of motor skills lead to permanent changes in brain synapses (Dzedzickis et al., 2020). We believe that, similar to archery (Gu et al., 2022), football (Li et al., 2022), and other sports, long-term professional dragon boat training promotes sustained activation of the athlete’s dorsal attention network. During the training sessions in this study, the athletes were more focused and therefore required less cognitive effort to achieve the desired nervous system state.

The results of EEG microstate analysis after moderate intensity training showed that the duration of microstates classes C and D and the contribution of microstate class C significantly increased and the frequency of microstate class D significantly decreased in the novice group after moderate intensity training, which is more consistent with the findings of Spring et al. (2017), where the mean duration and contribution of microstate class C were elevated after longer endurance cycling. In addition, in a cycling experiment conducted at an exercise intensity near the anaerobic threshold for about 30 min, it was found that the organism was still unable to completely recover to quiet levels 1 h after exercise, and the mean duration of microstate class C was elevated for 1 h after exercise (Spring et al., 2018). Previous studies have shown that regions associated with microstate class C activation, such as the anterior insula, anterior cingulate gyrus, and top of the frontal lobe, play an important role in the transition between the central executive network and the default mode network (Sridharan et al., 2008), and are also key parts associated with cognition, emotion, and motivation. At the same time, the cingulate cortex network is associated with the maintenance of alertness and its enhanced functional connectivity characterizes increased cognitive abilities (Sadaghiani and D'Esposito, 2015). The central nervous system and musculoskeletal muscle system are related (Enders and Nigg, 2016), and muscle fatigue causes reduced cortical muscle coupling (Siemionow et al., 2010). Exercise fatigue leads to increased EMG signals along with increased activation of brain regions such as ipsilateral sensorimotor areas and cingulate gyrus (Liu et al., 2003), and studies have also shown that the convexity network corresponding to microstate class C is associated with muscle fatigue (Spring et al., 2017). Meanwhile, the cardio-cerebral response has received attention, and the autonomic nervous system is more widely distributed in the body, and involved in cardiac regulation, blood circulation, gastrointestinal function, and thermoregulation (Benarroch, 2020). The novice group in this study showed significant changes in microstate class C after exercise, probably due to the development of exercise fatigue as a result of intense simulated competition. This suggests that novice athletes should not pursue too much training in the daily training process, because high-intensity exercise training is more likely to lead to fatigue. There is often a certain gap between the physical function of novice and expert athletes, so novice athletes should follow the principle of step by step in the formulation of training programs to prevent the accumulation of sports fatigue and sports injuries. But expert athletes in the long-term training, the body has been able to withstand a large load of training volume, so it can be appropriate to increase the intensity of exercise, improve the special ability.

In terms of transition probability, the results of this study showed that the transfer probabilities of A to D, C to D, and D to A were significantly higher in the expert group than in the novice group in the quiet state pre-exercise, and post-exercise, the transition probabilities of A to B were significantly higher in the expert group than in the novice group, while the transition probabilities of D to C and C to D were significantly lower than in the novice group, and the changes pre- and post-exercise may be because the expert group put less effort into performing the same task but could achieve better performance. In addition, the results showed that the novice group was more likely to switch microstate class from B and D to C and from C to D after movement, suggesting that other networks are more likely to shift to the convex network, while the convex network is likely to shift to the dorsal attention network, similar to the study by Spring et al. (2018). The expert group in this study had an elevated probability of switching from C to D only, suggesting that moderate-intensity training is more likely to activate the attentional network of transferred athletes and that the neural state of the brain of excellent archers is preferentially shifted to the dorsal attentional system (Gu et al., 2022). Microstate class D has not received much attention in the current research, but our study shows that long-term motor training has an important effect on microstate class D associated with attentional networks, and its role needs to be studied and explored in depth in the future.

## 5. Conclusion

In this study, the power spectral density values of the professional dragon boat athletes after long-term training in the *δ*, *α*1, *α*2, and *β*1 bands are higher than those of the amateur group. After acute moderate intensity, the power spectral density of *α*2, *β*1, and *β*2 bands in the professional group was higher than that in the amateur group. The microstate analysis shows that long-term training has a more significant impact on the microstate type D of dragon boat athletes, while acute medium-intensity training has a more prominent impact on the microstate C and D of dragon boat athletes. The results of this study suggest that sustained motor skill learning facilitates the development of internal plasticity in the brain, and that acute moderate-intensity exercise activates the cerebral cortex, stimulating nerve cell excitation and thus improving cognitive function. At the same time, the microstate analysis results further confirm the differences in the brain attention network between expert and novice athletes and the important role of the attention network associated with microstate class C in sports. In the future, EEG microstate analysis can be applied more in the field of locomotion to explore the changes brought about by locomotion on macroscopic scales. At the same time, it is hoped that EEG analysis can help dragon boat sport to formulate sports training programs more scientifically.

In this study, physiological measurement was used to initially attempt to explore the changes in brain mechanisms in resting states of dragon boat racers pre and post-exercise at different levels, but there were some shortcomings in the study. In terms of the study population, the selection of expert athletes in this study was limited to the college student, and the higher level dragon boat players in the national team could be selected later. The subjects recruited in this study are all male. What are the brain activities of female students after dragon boat training and whether there are differences between men and women and other questions worthy of further exploration. Since the spatial resolution of EEG is insufficient, we can also try to use near-infrared and nuclear magnetic resonance technology to monitor dragon boat athletes. During the data collection, because the athletes washed their hair immediately after exercise and then collected the data, the athletes were still in the recovery stage. At this time, due to factors such as sweating and fast breathing rate, some artifacts may be generated in the data, EEG signal itself is also unstable, easy to be affected by emotions and external factors, which may have a certain impact on the final result. In terms of research methods, microstate analysis, although capable of quantifying cognitive states, has been relatively little applied in the motor domain and its physiological significance needs further validation. In addition, this study is mainly looking for the changes of the brain neural mechanism in the resting state. With the development of portable sports EEG, we can also try to explore the brain activity state of dragon boat athletes during rowing in the future. And test the executive function of athletes with more mature experimental paradigm.

## Data availability statement

The original contributions presented in the study are included in the article/Supplementary material, further inquiries can be directed to the corresponding author.

## Ethics statement

The studies involving human participants were reviewed and approved by Academic Committee of Shaanxi Normal University. The patients/participants provided their written informed consent to participate in this study. Written informed consent was obtained from the individual(s) for the publication of any potentially identifiable images or data included in this article.

## Author contributions

QW and AC were involved in the design of the study, data collection and data analysis, manuscript finalization, and drafted the manuscript. QW, AC, and CW took the lead in the manuscript finalization and submission. YZ, WZ, YC, HJ, CS, and BS were involved in data collection and the design of the study. All authors contributed to the article and approved the submitted version.

## Funding

This research was supported by the Shanghai Education Commission - Shanghai Educational Science Research Project (no. C2021198), Signature Achievement Project of Sports School in Shaanxi Normal University (nos. 2022AA002 and 2022AA003).

## Conflict of interest

The authors declare that the research was conducted in the absence of any commercial or financial relationships that could be construed as a potential conflict of interest.

## Publisher’s note

All claims expressed in this article are solely those of the authors and do not necessarily represent those of their affiliated organizations, or those of the publisher, the editors and the reviewers. Any product that may be evaluated in this article, or claim that may be made by its manufacturer, is not guaranteed or endorsed by the publisher.
