# Supplementary figures and images for: Characteristics of changes in the functional status of the brain before and after 1,000 m all-out paddling for different levels of dragon boat athletes
Source: Front Psychol. 2023 May 23;14:1109949. doi: 10.3389/fpsyg.2023.1109949 (PMC10243504; doi:10.3389/fpsyg.2023.1109949)

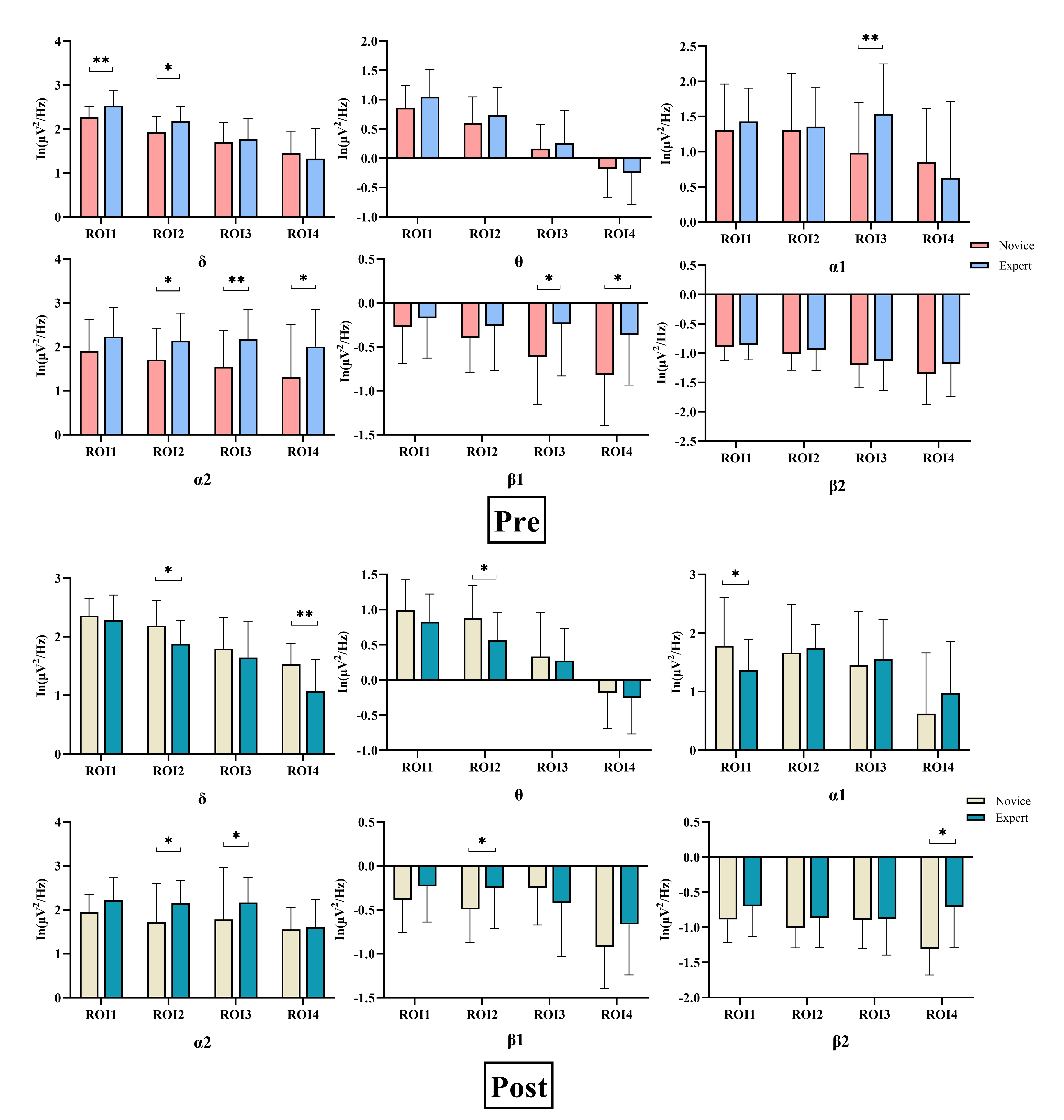

Supplement: Supplementary file 1 [file Image_1.PNG]

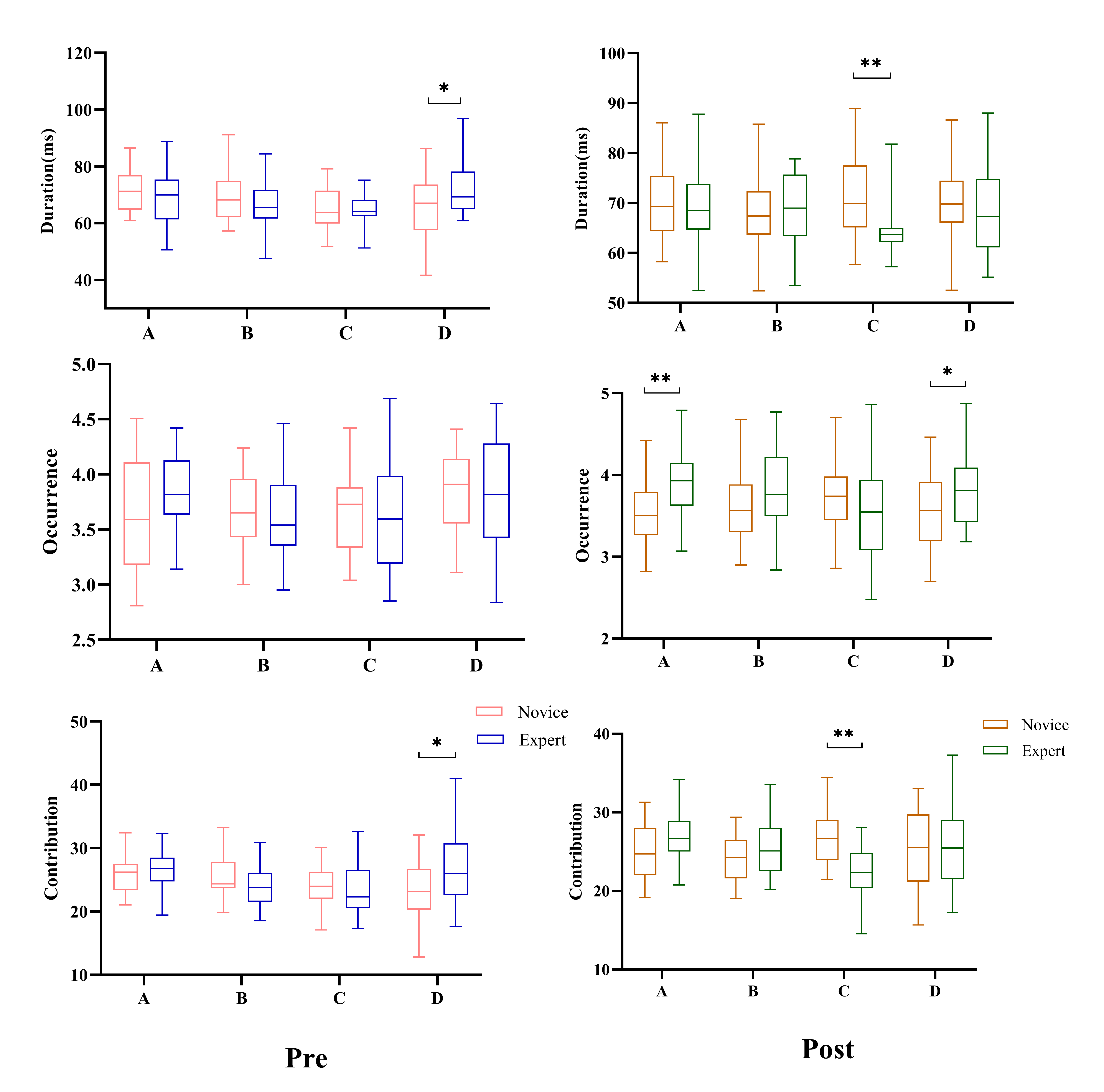

Supplement: Supplementary file 2 [file Image_2.PNG]
